# Overview of Ten Child Mental Health Clinical Outcome Measures: Testing of Psychometric Properties with Diverse Client Populations in the U.S.

**DOI:** 10.1007/s10488-021-01157-z

**Published:** 2021-09-05

**Authors:** F. Alethea Marti, Nadereh Pourat, Christopher Lee, Bonnie T. Zima

**Affiliations:** 1 Center for Health Services and Society, UCLA-Semel Institute for Neuroscience and Human Behavior, 10920 Wilshire Blvd. #300, Los Angeles, CA 90024 USA; 2 UCLA Center for Health Policy Research, UCLA Fielding School of Public Health, 10960 Wilshire Blvd #1550, Los Angeles, CA 90024 USA; 3 Santa Clara County Public Health Department, 976 Lenzen Avenue, San Jose, CA 95126 USA; 4 UCLA School of Dentistry, 714 Tiverton Ave., Los Angeles, CA 90095 USA

**Keywords:** Child mental health, Clinical outcome measures, Disparities in care, Psychometric properties, Quality monitoring

## Abstract

While many standardized assessment measures exist to track child mental health treatment outcomes, the degree to which such tools have been adequately tested for reliability and validity across race, ethnicity, and class is uneven. This paper examines the corpus of published tests of psychometric properties for the ten standardized measures used in U.S. child outpatient care, with focus on breadth of testing across these domains. Our goal is to assist care providers, researchers, and legislators in understanding how cultural mismatch impacts measurement accuracy and how to select tools appropriate to the characteristics of their client populations. We also highlight avenues of needed research for measures that are in common use. The list of measures was compiled from (1) U.S. state Department of Mental Health websites; (2) a survey of California county behavioral health agency directors; and (3) exploratory literature scans of published research. Ten measures met inclusion criteria; for each one a systematic review of psychometrics literature was conducted. Diversity of participant research samples was examined as well as differences in reliability and validity by gender, race or ethnicity, and socio-economic class. All measures showed adequate reliability and validity, however half lacked diverse testing across all three domains and all lacked testing with Asian American/Pacific Islander and Native American children. ASEBA, PSC, and SDQ had the broadest testing.

**Supplementary Information:**

The online version contains supplementary material available at 10.1007/s10488-021-01157-z.

While many standardized assessment tools have been developed to track child mental health treatment outcomes on the individual and aggregate level, the degree of testing for reliability and validity across race, ethnicity and class is uneven. Nearly two thirds (62.1%) of children who received treatment through the national Children’s Mental Health Initiative (CMHI) from 2013 to 2017 were racial or ethnic minorities or biracial, and 71.3% came from families at or below the federal poverty threshold (SAMHSA, 2020). For this reason, it is crucial that clinicians have the information to select measures with proven reliability and validity for their clients.

This paper reviews the published psychometrics literature on the most commonly used standardized outcome measures in use for U.S. outpatient community child mental health care, with two aims:

1. To assist clinicians and policy makers in making informed decisions when selecting a standardized outcome measurement system.
2. To highlight needed avenues of additional testing for commonly used measures, to ensure suitability for diverse client populations.

To these ends, this paper presents a summary of the psychometrics tests for each of the investigated measures, focusing on disparities across gender, class, and race and ethnicity, followed by a comparison of the entire corpus to examine which populations are systematically overlooked across studies. We also briefly discuss the importance of taking into account differences in community background (whether these be race or ethnic group, class, or immigrant culture) and highlight specific ways such differences can impact measurement accuracy, as well as recommended readings for clinicians on the topic of culturally congruent care.

The literature scans for this review were conducted as part of a contracted project with the California Department of Health Care Services (DHCS).

## Background

Reducing disparities in the access to, and quality of, child mental health care has long been identified as a national priority area (Perou et al., 2013; US Department of Health and Human Services et al., 2000). The U.S. Department of Health and Human Services’ National Quality Strategy envisions that quality improvement is driven by linking recommended care processes to meaningful clinical outcomes, as well as aligning financial incentives to promote effective care (AHRQ, 2016). The Patient Protection and Affordable Care Act was passed in 2010 (42 U.S.C. § 18,001 et seq.). Although California legislated a mandate in 2011 to develop a performance outcome system for children (SB 1009; California Legislative Analyst’s Office, 2011), the state’s development of data infrastructures to monitor quality and detect disparities have considerably lagged behind national recommendations (Gardner & Kelleher, 2017; Glied et al., 2015; Patel et al., 2015; Pincus, 2012; Zima et al., 2013).

In 2016, the California Department of Health Care Services (DHCS) contracted our university to help them address the question, “What is the best statewide approach to evaluate functional status for children/youth that are served by the California public specialty mental health service system?” (DHCS, 2015, p. 6). To assist the CA DHCS in developing their outcome monitoring infrastructure, we created a list of all standardized measures in common use for tracking overall child mental health outcomes in the U.S., and ranked them on nine minimum criteria (Pourat et al., 2017; Zima et al., 2019). In this paper, we will lay out the findings from two rounds of systematic literature scans, and examine the breadth and diversity of psychometric testing on each candidate measure, namely: (1) published evidence for its use as a clinical outcome measure; (2) its psychometric properties and variation among diverse study populations; and (3) whether the existing published evidence adequately included children of diverse genders, classes, ethnicities and races. Details about demographics, sample size, etc., for each of the cited studies can be found in the tables. Results are divided into two sections: first the analyses of individual measures, then an aggregate analysis of the entire corpus for systematic patterns and gaps.

The Discussion section examines the significance of these results, particularly the systematic under-representation of Native Americans and Asian Americans. We also discuss the importance of clinicians’ having an understanding of cultural and linguistic differences across class, race, and ethnicity (in addition to cultural differences for immigrant families), and guide clinicians in recognizing specific types of misreporting that can occur if a measure is not properly developed for a particular population. Finally, we examine the pros and cons of three possible solutions: creating adjusted scoring guides; using internationally developed questionnaires for immigrant children; and developing measures specifically for under-represented populations.

## Methods

### Compiling a List of Candidate Measures

The first step in this project was to create a pool of all eligible measures in common use in the U.S. that might meet the DHCS’s needs, after which we would research each individual measure more closely. We conducted three initial investigations: (1) an exploratory 5-year literature scan; (2) an environmental scan of U.S. state Department of Mental Health agency websites, and (3) two statewide California surveys. Additionally, (4) DHCS requested information on four measures that had been recommended to them by other sources. Table 1 lists which criteria were met for each measure.

**Table 1:** Candidate child mental health outcome measures by selection criteria

Measure^a^	Psychometrics articles^b^	Inclusion criteria			
		Exploratory lit. scan^c^	CA county surveys	DMH websites	DCHS request
Pediatric Symptom Checklist (PSC)	23	2		✓	
Achenbach System of Empirically Based Assessment^d^ (ASEBA)	22	21 ✓	✓		
Strengths & Difficulties Questionnaire (SDQ)	12	13 ✓		✓	
Child and Adolescent Functional Assessment Scale^e^ (CAFAS)	4	2	✓	✓	✓
Children’s Global Assessment Scale (CGAS)	4	14^f^ ✓			
Child & Adolescent Needs & Strengths (CANS)	3	3	✓	✓	✓
Youth Outcome Questionnaire (Y-OQ)	3	4 ✓	✓	✓	
Ohio Youth Problem, Functioning and Satisfaction Scales (Ohio)	2	4 ✓		✓	✓
Treatment Outcome Package (TOP)	1	–			✓
Clinical Global Impressions Scale (CGI)	–	8 ✓			

*DHCS* = California Department of Healthcare Services; *DMH* = state Department of Mental Health. Check-mark indicates that measure met inclusion criteria for a given source: Exploratory scan: appeared in at least 3 studies; see Table 2 for details; CA county survey: reported use in at least 2 California counties; DMH websites: recommended by at least 2 State DMH agency webpages; DHCS request: At the beginning of the project, DHCS expressed interest in four measures; all except TOP also met other inclusion criteria

^a^Total count includes subcomponents, informant-specific report versions, age-specific versions, and treatment planning versions of measure

^b^Systematic literature scans to describe psychometric properties and use in diverse populations

^c^Exploratory scan (2010–2016) to identify use of clinical outcome measures in community-based treatment settings

^d^Count includes the Child Behavior Check List (CBCL); Youth Self Report (YSR) and Teacher Report Form (TRF)

^e^Count also includes the Preschool and Early Childhood Functional Assessment Scale (PECFAS)

^f^Count does not include an additional 7 studies using GAS/GAF (Global Assessment Scale/Global Assessment of Functioning) with children

#### Exploratory Literature Scan

Systematic searches of PubMed, PsycInfo, and Scopus were conducted for peer-reviewed journal articles published between January 2010 and December 2015, with English language abstracts, that examined children ages 0–18 years in U.S. community-based outpatient care.^1^ (Further details on article inclusion and exclusion criteria are in Supplemental Table 1, the 5-year cutoff was deliberately chosen to capture measures in current use). A list was compiled of all the standardized measures that were used in these studies as data collection tools to track outcomes, resulting in approximately 225 child clinical outcome measures in 127 unique articles. Thirty-four of these measures appeared in three or more articles.^2^ Of these, twenty-one were specific to a single diagnosis or condition (e.g. ADHD), three were general health or quality of life measures, and three were excluded for other reasons,^3^ leaving seven candidate measures.

#### Measures in Common Use (Nationally or in California) or of Interest to the DHCS

An environmental scan of state Department of Mental Health agency websites was conducted to determine which measures were in common use across the U.S. Thirty-five states listed at least one standardized assessment measure, for a total of 36 different measures (Pourat et al., 2016b).

To determine which measures were in common use in California, we conducted a statewide survey of county behavioral health agency directors (56 counties), and a second convenience sample of outpatient clinic staff (21 responses) which yielded seven eligible measures that were used in more than one county (Pourat et al., 2016a). Finally, the DHCS Subject Matter Experts team requested we also investigate four measures that had been recommended to them (three of which also appeared in the county and state lists).

#### Required Scope of Candidate Measures

To align with the priorities of our DHCS agency partners, the list was narrowed to measures that had the following characteristics: (1) track overall behavioral or emotional health (i.e. not specific to a single diagnosis such as depression); (2) are designed for children 5–16 years; (3) have been normed or tested with children in the U.S.; and (4) produce quantifiable scores that can be used to compare treatment outcomes of different patients, or can be aggregated to compare the quality of care of different service provides.

The final list consisted of all measures meeting those criteria that also met at least one of the following use criteria: (1) appeared in at least 3 studies in the exploratory literature scan (7 measures); (2) was reported by at least 2 California counties (4 measures); or (3) was recommended on at least 2 state DMH webpages (6 measures). One measure on the DHCS interest list did not meet any of the other criteria (TOP); it was also included. This yielded a total of 10 measures flagged for further investigation, listed in Table 1.

### Examination of Psychometric Properties and Capacity to Identify Disparities

After the candidate list was compiled, ten systematic literature reviews (one for each measure) were conducted to examine psychometric properties and suitability for diverse communities. For each measure, a Web of Science search was made of published articles with English language abstracts from the measure’s initial development through March 2019, that tested reliability and/or validity with children under 18 years of age for either symptoms or functioning.^4^ (Further details on article inclusion and exclusion criteria are in Supplemental Table 2) Literature reviews and meta-analyses were manually examined for additional citations. Further citations were obtained from measure developers’ or vendors’ webpages (if they existed) as well as from articles recommended for inclusion by a DHCS-selected team of Subject Matter Experts.

Studies that focused on specific demographics (e.g. by ethnicity or socioeconomic status) were included, as well as studies focusing on populations that might be recipients of outpatient care in California (e.g. immigrants or adopted children). Studies that used non-English versions of the measure were included if they used an independent metric to test reliability and validity (i.e. were not simply comparing a translation to the English language original).

We examined the characteristics of the research participants in each study and across the entire corpus, as well as noting reported differences in psychometric properties by race or ethnicity, gender, and class or SES. For ethnicity and gender, the researchers’ own categories were used. To determine class diversity, we looked for: explicit mention of SES or household income, more general class labels (e.g. “working-class," “upper middle class,” “poor”), eligibility for financial services or other aid (e.g. Medicaid or free school lunches), or enrollment in programs specifically designed for low-income families (e.g. HeadStart).

## Results for Individual Measures

The final list of candidate measures by selection criteria are summarized in Table 1. The candidate measures were Achenbach System of Empirically Based Assessment (ASEBA); Child and Adolescent Functional Assessment Scale (CAFAS); Child and Adolescent Needs and Strengths (CANS); Children’s Global Assessment Scale (CGAS); Clinical Global Impressions Scale (CGI); Ohio Youth Problem, Functioning and Satisfaction Scales (Ohio); Pediatric Symptom Checklist (PSC); Strengths and Difficulties Questionnaire (SDQ); Treatment Outcome Package (TOP); and Youth Outcome Questionnaire (Y-OQ).

### Use as a Clinical Outcome Measure in Community-based Mental Health Programs

Findings from the exploratory literature scan are summarized in Table 2. The three measures most frequently used to track clinical outcomes among children receiving community-based mental health care were ASEBA (21 studies),^5^ CGAS (14 studies), and SDQ (13 studies). Five measures were only used in the U.S. (CAFAS, CANS, Ohio, PSC, and Y-OQ), while four were also used internationally (ASEBA, CGAS, CGI, and SDQ). TOP was added to the list of measures to investigate at the request of DHCS, however it did not appear in the literature scan. One fifth (13 of 57) of the studies combined multiple candidate measures, most frequently CGAS with either SDQ (4 studies) or CGI (3 studies). Other measures used in combination were: CGI (6 of 8 studies), SDQ (6 of 13), PSC (2 of 2), ASEBA (3 of 21), CAFAS (1 of 2), and Ohio (1 of 4). All measures were applied to children with a diverse range of mental health conditions including: general use across psychiatric conditions (18 studies), broad categories such as behavioral or emotional problems (6 studies) or trauma (7 studies); or specific diagnoses such as anxiety (7 studies) or ADHD (5 studies).

**Table 2:** Use of candidate measures to assess child mental health outcomes in community-based programs

Measure & references *(Bold = study listed under multiple measures)*	Age range & sample sizes	Target condition	Treatment setting	Other measures	Follow-up intervals
**ASEBA:** (21 articles) *CBCL (parent respondent)* **a = Balottin et al. (**2014**);** b = Cohen et al. (2011); c = Dorsey et al. (2014); d = Eslinger et al. (2015); e = Liber et al. (2010); f = Liotta et al. (2015); g = McCrae et al. (2010); h = Mittler et al. (2014); i = Painter (2012); j = Palma et al. (2015); **k = Shapiro et al. (**2012**);** l = Tan and Martin (2015); m = Tsai and Ray (2011); n = Vishnevsky et al. (2012) *CBCL only some sub-scales* o = Southam-Gerow et al. (2010); **p = Storch et al. (**2015**)** CBCL (parent) YSR (youth) & BPC* q = Dour et al. (2013) *TRF (teacher respondent)* r = Cantos and Gries (2010) *CBCL and TRF* s = Overbeek et al. (2014); t = Rothmann et al. (2014) *CBCL and YSR* u = Misurell et al. (2014)	2–18 years 33–1790	Any^r,m^ primary psychiatric diagnosis^l^ ADD/ADHD^j,t^ Anxiety^e,o,p^ Child sexual abuse^f,g,u^ Disruptive behaviors^k^ Emotional disorders/disturbances^h,i,n^ Idiopathic headaches^a^ Interparental violence^b,s^ Maltreatment^g^ PTSD or trauma^b–d^ “Sudden gain” between weekly therapy sessions^1^ *Other characteristics:* children in foster care^c,r^	Neuropsychiatry outpatient service^a^ Interdisciplinary neuropsychological child care center^j^ Community MH center^h,o,p^ for children and adolescents^k,l^ Specialty center for stress trauma,^d^ IPV^s^ or child abuse/maltreatment^f^ University based counseling clinic^m^ System of care^i,n^ Outpatient^E^ or hospital outpatient^u^ Referrals from foster care,^r^ child welfare^c,g^; community women’s shelter^b^ Not specified,^t^ treated by therapists from outpatient clinical service orgs (clinics/schools)^q^ Sample compared with nonclinical control group^h^ *Countries:* Australia,^l^ Brazil,^j^ Germany,^t^ Israel,^h^ Italy,^a^ Netherlands,^e,s^ USA,^b,c,f,g,k,m,n,p,q,r,u^ not specified but probably USA^d,i,o^ *Translations:* German,^t^ Hebrew,^h^ Dutch^e,s^; not specified but probably Portuguese,^j^ Italian^a^; bilingual evaluator but language not specified^i^; not specified, study used existing archival records^m^ *Note: information from abstract as full article was not in English*^t^	CGI^a,p^ Ohio^k^	*Baseline & follow-up:* 1 week^e^; 3 months^s^; 6 months^a,h,i,n,r^; 18 months^g^; 4 years^j^; treatment midpoint^p^; *Baseline & end of treatment:* 5 weeks^l^; 8 weeks^b^; 12 weeks^p^; variable time^c,d,m,o,u^; not specified^qt^ *After end of treatment:* 1 weeks; 1 month^p^; < 2 months^k^; 3 months^c,l^; 6 months^s^; variable time^1^; approximately 18 months after baseline^q^; *Other:* Within 1 month of start & end^f^; scores from archival record, variable follow-up times^m^
**CAFAS:** (2 articles) *Parent respondent, school-age version* **a = Bruns et al. (**2015**);** b = Mueller et al. (2010)	6–17 years 81–2171	Any^b^ Serious emotional disorder^a^	Division of child and family services or private MH agency^a^ Child and adolescent MH system^*b*^ *Countries: *USA^a,b^	SDQ^a^	Baseline, 6 months & 12 months^a^ Data are from clinical record, interval not specified^b^
**CANS:** (3 articles) *Clinician respondent* a = Accomazzo et al. (2015); b = Dunleavy and Leon (2011); c = Radigan and Wang (2013)	4–19 years 77–793	Any^c^ Antisocial behavior^b^ Emotional, behavioral & environmental issues^a^	Urban publicly funded behavioral health system^a^ Community-based system of care^b^ State mental health service providers^c^ *Countries:* USA^a−c^ *Note: all studies used CANS data that was collected as part of clinical practice*	None	Baseline & every 6 months^a,b^ Baseline & discharge^b,c^
**CGAS**: (14 articles) *Clinician respondent* **a = Clark et al. (**2014**);** **b = Clarke et al. (**2015**);** **c = De Souza et al. (**2013**);** d = Duffy and Skeldon (2014); e = Foa et al. (2013); **f = Hansen et al. (**2015**);** g = Lundh et al. (2013); **h = Murphy et al. (**2015**);** **i = Murphy et al. (**2012**);** **j = Nilsen et al. (**2015**);** k = Stefanovics et al. (2014); **l = Tse et al. (**2015**);** m = West et al. (2014); **n = Wolpert et al. (**2012**)**	4–20 years 30–12,613	Any^d,h,i,n^ mild/moderate MH concerns^a^ Abuse, maltreatment or neglect^k^ ADHD^g,l^ Anxiety^c,f^/mood disorders^f^ Depression^b^ Emotional disorders^j^ Insomnia^b^ Pediatric bipolar disorder^m^ PTSD/sexual abuse^e^	Community MH clinic/service^e^, CAMHS^a,d,g,j,n^ or CYMHS^f^ hospital-based outpatient psychiatry services^h,i^ Outpatient child psychiatry^i,m^ Community-based rehabilitation^k^ Telemental health^l^ Not specified^c^ but referred through hospital medical records^b^ or community^b^ *Countries:* Australia^f^ Brazil^c,k^ New Zealand^a^ Norway^j^ Scotland^d^ Sweden (Swedish)^g^ USA^b,e,h,i,l^ UK^n^, not specified but probably USA^m^ *Translations:* Swedish^g^; not specified but probably Norwegian ^j^ Portuguese^c,k^ *Note: five studies used CGAS scores that were collected as part of clinical practice*^f–i,n^	CGI^b,c,l^ PSC^h,l^ SDQ^a,f,j,n^	*Baseline & follow-up:* 4 weeks^m^; 7 weeks^c^; 12 weeks (but not baseline)^b^; 25 weeks^l^; 3 months^f,h–k^; 6 months^j,k^; mid and post treatment but time not specified^e^ *Baseline & end of treatment:* At < 1 months^g^; at 4–8 months^n^; variable^a,d^ * After end of treatment:* 3 months^e^, 6 months^e,m^, 12 months^e^
**CGI:** (8 articles) *Clinician respondent* **a = Balottin et al. (**2014**);** **b = Clarke et al. (**2015**);** **c = De Souza et al. (**2013**);** d = Salloum et al. (2014); **e = Storch et al. (**2015**);** **f = Thirlwall et al. (**2013**)** *“Improvement” component only* g = Creswell et al. (2010); **h = Tse et al. (**2015**)**	3–20 years 5–100	ADHD^h^ Anxiety^c,e–g^ Depression^b^ Idiopathic headaches^a^ Insomnia^b^ Post Tramatic Stress symptoms (PTSS) + trauma^d^	Neuropsychiatry outpatient service^a^ Outpatient community MH center,^e^ CAMHS^g^ or CAMHS connected child anxiety/clinic^f^ Telemental health^h^ Not specified^c,d^ but referred through hospital medical records^b^ and community^b^ *Countries:* Brazil^c^ Italy^a^ UK^f,g^ USA^b,e,h^ not specified but probably USA^d^ *Translations:* not specified but probably Italian^a^ Portuguese^c^	ASEBA^a,e^ CGAS^b,c,h^ SDQ^f^	*Baseline & follow-up:* 7 weeks^c^; 12 weeks^e^ (but not baseline^b^); 25 weeks^h**^; 6 months^a,f^; variable^d^ *Baseline & end of treatment only:* 8 weeks^f^ *After end of treatment:* 4 weeks^g^; 1 months^e^; 3 months^d^; 6 months^f^
**Ohio:** (4 articles) *Parent respondent* **a = Shapiro et al. (**2012**);** b = Cook et al. (2014) *Parent & child respondents* c = Karpenko and Owens (2013) *Clinic staff respondent* d = Tucker et al. (2013)	3–21 years 67–1135	Any^c,d^ Disruptive behaviors^a,b^	Intensive outpatient^b^ Community clinic^a^ or MH centers^c,d^ *Countries:* USA^a−d^	ASEBA^a^	*Baseline & follow-up:* Weekly^b^, 3 months^c^ Baseline and at^d^ or 2 months after^a^ discharge
**PSC:** (2 articles) *Parent respondent* **a = Murphy et al. (**2015**);** **b = Murphy et al. (**2012**)**	0–17 years 106–531	Any^a,b^	Hospital—outpatient child and/or adolescent psychiatry^a,b^ *Countries:* USA^a,b^	CGAS^a,b^	*Baseline and follow up:* 3 months^a,c^
**SDQ:** (13 articles) *Parent and child respondents* **a = Hansen et al. (**2015**);** **b = Nilsen et al. (**2015**);** **c = Thirlwall et al. (**2013**);** **d = Wolpert et al. (**2012**)** *Parent, child & teacher* e = Dura-Vila et al. (2013) *Parent only* f = Grip et al. (2012); g = Grip et al. (2013); h = O’Donnell et al. (2014) *Child only* **i = Clark et al. (**2014**);** j = Jensen et al. (2014) *Respondent unknown* **k = Bruns et al. (**2015**);** l = Coren et al. (2013); m = Foreman and Morton (2011)	3–18 years 11–583	Any^d,e^ mild/moderate^i^ ADHD^m^ Anxiety^a,c,f^/mood^a^ disorder Child sexual abuse^l^ Emotional disorder^b,k^ Exposure to intimate partner violence^f,g^ PTSD^h^ or trauma^j^	Safe & Secure Network (outpatient therapy)^l^ Community-based service or program^e,f,g,j^; CAMHS^b,d,i,m^ or CMYHS^a^; or CAMHS-connected specialty clinic^c^ Referral by service organization^h^ Women’s shelter^g^ Compared state versus private MH services^k^ *Other demographics:* children of refugee/asylum-seeking families^e^ *Countries: *Australia^a^, New Zealand^i^ Norway^b,j^ Sweden^f,g^ Tanzania^h^; UK^c,d,e,l,m^ USA^k^ Translations: *** Arabic,^e^ Albanian,^e^ Kiswahili,^h^ Kurdish,^e^ Norwegian^b,j^; Somali,^e^ Swedish^f,g^	CAFAS^k^ CGAS^a,b,d,i^ CGI^c^	*Baseline & follow-up:* Monthly^a^; 6 months^b,f,g,j,k^; 12 months^l^; 4–6 years^m^; time interval varied^b^ *Baseline & end of treatment only:* At 12 weeks^h^; 4–8 months^d^; < 12 months^l^; time interval varied^e,i,j^ *After end of treatment:* 3 and 12 months^h^; 6months^c^
**Y-OQ:** (4 articles) *Parent respondent* a = Warren et al. (2010); b = Warren et al. (2012) *Parent and youth respondents* c = Cannon et al. (2010); d = Warren and Salazar (2015)	4–17 years 104–953 in community clinics^a−d^ 1762–3705 in private care^a−c^	Any^a,b,d^ At risk for treatment failure^c^ (*Two articles used the same archival data set.*^*a,b*^*)*	Community mental health center or system^a−d^ Compared to: – Commercial regional health center corporation^c^ – Private managed care organization^a,b^ *Countries:* USA^a−d^	None	*Baseline & regular intervals:* 3 weeks, 2 months, 4 months, 6 months^d^ Data were obtained from clinic records; time intervals and number of follow-ups varied per patient^a−c^

Other measures column only includes other measures examined in this paper. Follow-up intervals are between consecutive uses (e.g. a study applying a measure at 6, 12, and 18 months has a 6 month interval)

* *BPC* = Brief Problem Checklist, a 3rd party measure adapted from CBCL/YSR

** Measure was administered at each session, however the published article only compared the scores at baseline and 25 weeks

*** Dura-Vila et al. (2013) (labeled as reference “e”) did not specify whether they used a translated SDQ or had an interpreter verbally translate the English measure for the family. Since SDQ is available in all of the listed languages, we will assume the former

All ten measures were designed for wide age ranges and covered at minimum 5–18 years. Despite this, over one quarter of the studies (15 of 57) used a measure for children outside the recommended age range.

Follow-up intervals varied extensively and did not consistently correspond to the measure’s recommended use. One fifth (12) of the studies used archival data from existing clinic records, illustrating the feasibility of using the measure in clinical practice, but also indicating that clinics do not always track their clients’ outcomes at consistent, regular intervals. Similarly, one third (21 studies) administered the measure only at intake and end of treatment or patient discharge, which led to variation in episode of care across their data set.

### Sample Diversity and Psychometric Properties of Candidate Measures

The following sections summarize the diversity of study samples and overall psychometrics for each individual measure. Sample characteristics for each study are summarized in Table 3. Measures are listed in order of number of published studies. Most had less than five studies testing reliability and validity with U.S. children, however PSC (23 studies), ASEBA (22) and SDQ (12) were more extensively tested.

**Table 3:** Demographic characteristics among study samples for studies reporting psychometric properties of candidate measures

References	System identified children	Non diagnosed children (“Community” or “control” group)	Other relevant categorizations
*(Bold = coauthored by a developer of the measure)*	*(Receiving MH care or referred for emotional or behavioral problems)*	*(Sampled from community, school, or pediatric/well-child visits)*	
**ASEBA**—developed in 1983 (Achenbach & Edelbrock, 1983)			
*23 studies (1 by developers*) a = Bird (1987a) (2 samples) b = Dedrick et al. (1997); c = Dutra et al. (2004); d = Ebesutani et al. (2010); e = Ebesutani et al. (2011); f = Hogez and McKay (1986); g = Jastrowski Mano et al. (2009); h = Jensen et al. (1993, 1996) (same data set); i = Knelpley et al. (2019); j = Konold et al. (2004); k = Lambert et al. (2002); l = Nakamura et al. (2009); m = Nelson et al. (2002); n = Reed and Edelbrock 1983 (2 samples); o = Rescorla et al. (2017); p = Rishel et al. (2005); q = Rubio-Stipec et al. (1990); r = Salcedo et al. (2018); s = Sheldrick et al. (2015) (2 samples); t = Song et al. (1994); u = Tharinger et al. (1986); v = Tyson et al. (2011)	*Ages:* 4–19 years *Sample sizes:* ^a–e,i,k,l,o,p,r,t^ 191 to 1,605 *Gender:* over 60% male^b,d,e,k,l,p,r^; over 60% female^T^; balanced^a,c,i,o^ *Ethnicity:* over 70% White or Caucasian^b,c,i,p,t^; 70–100% African American^k,r^; Puerto Rican^a^; primarily multi-ethnic^l^ and White^d,e^; 14% Asian^l^; not specified^o^ *SES/Class:* mainly middle/upper-middle class^c,t^; mainly low income^a,p^ with annual household income mostly < $40k^d,e,r^; not specified^b,i,k,l,o^ *Translations:* Spanish^a^	*Ages:* 54 months–16 years *Sample sizes:* 15–777^a,f,j,n,q^; * Behavioral problems group:* 15–910^g,m,n,u^ *Gender:* all male^f,n^; balanced gender^a,j^; not specified (3 samples)^j,s^ but stratified by age/sex^q^; *Behav probs*: all male^n,u^; 57–61% male^g,m^ *Ethnicity:* over 80% White^j,n^; all Puerto Rican^a,q^; not specified (3 samples)^f,s^; *Behav probs*: 63% Caucasian, 24% African American^m^; all but one White^n^; all African American^g^; not specified^u^ *SES/Class:* 15% below poverty level^j^; mainly middle class^n^ diverse range^a^; not specified (4 samples)^f,s,q^; *Behav probs*: mainly middle/upper-middle class^n,u^; not specified^m^ but 23.4% did not finish high-school and 57% had some college education^g^ *Translations:* Spanish^a,q^	*Special needs adopted children*^*v*^ Ages: 6–18 years; Sample size*:* 910; Ethnicity: 67% White, (parents and children of same ethnicity); SES/Class: average annual income was $32.5k (low income) for African Americans and $60k for Whites (middle class); Gender not specified *Military family, portion of sample identified as needing special medical/educational resources*^*h*^ Age: 5–17 years; Sample size: 201; Gender: balanced; Ethnicity: 59% Caucasian, 27% African American; SES/Class: $25–30k/year
**CAFAS**—developed in 1989 [Hodges, (no date)]			
*4 studies (1 by developers)* a = Bates et al. (2006); b = Francis et al. (2012); c = Hodges and Wong (1996); d = Murphy et al. (1999)	*Ages: *3–17 years^b,c^ *Sample size:* 373–780^b,c^ *Gender: *over 60% male^b,c^ *Ethnicity:* 79% Caucasian, 20% African American^c^; 40% mixed, 17% Asian, 12% Caucasian^b^ *SES/Class: *Median annual income $18.5k (below FPL)^b^; modal household income $30–40k^c^ *Other demographics*: children of army personnel^c^	*Ages:* 3–5 years^d^ *Sample size:* 30 parent interviews^d^ *Gender:* 66% male^d^ *Ethnicity:* 63% Hispanic (both English and Spanish speakers), 26% Anglo^d^ *SES/Class: *mainly Medicaid recipients^d^ *Translation:* English and Spanish^d^; *Other demographics*: enrolled in HeadStart^d^	*No participants:* raters tested the measure on 20 fictional patient vignettes^c^ or graded individual questions^a^
**CANS**—developed in 1999 (Lyons, 1999)			
*3 studies (1 by developers)* a = Almadari and Kelber (2016) (CANS Short Form); **b = Anderson et al. (**2003**) (CANS-MH);** c = Kisiel et al. (2018) (CANS-Trauma)	*Ages:* 0–18 years^a−c^; *Sample size:* 60–257^a−c^; *Gender:* balanced^a^; over 58% male^b^; *Ethnicity:* 67–78% White, 10–24% Black^b,c^, 88% Hispanic^a^; *SES/Class:* all Medicaid eligible^b^, unknown^a,c^	*None*	
**CGAS**—developed in 1983 (Shaffer et al., 1983)			
*4 studies (2 by developers)* **a = Bird et al. (**, 1987b**) (2 samples);** b = Francis et al. (2012); c = Green et al. (1994); **d = Shaffer et al. (**1983**)**	*Ages: *4–17 years^a−d^ *Sample size:* 19–617^a−d^ *Gender:* samples stratified by age and gender^a^; 68–77% male^b,c^; all male^d^ *Ethnicity: *40% mixed, 17% Asian, 12% Caucasian^b^; Puerto Rican^a^; not specified^d,c^ *SES/Class:* low income families^b,c^; not specified^a,d^ *Translation:* Spanish^a^	*Ages: *4–16 years^a^ *Sample size*: 129^a^ *Gender:* samples stratified by age and gender^a^ *Ethnicity:* Puerto Rican^a^ *SES/Class:* not specified^a^ *Translation:* Spanish^a^	*No participants*:^d^ Raters examined 19 written case history vignettes
**CGI**—developed in 1976 (Guy, 1976)—*no studies*			
**OHIO**—developed in 2001 (Ogles et al., 2001)			
*2 studies (all by developers):* a = Dowell and Ogles (2008) (2 samples); b = Ogles et al. (2001) (6 samples)	*Ages: *8–17 years^a,b^ *Sample sizes:* 37–757^a,b^ *Gender: *63–70% male (3 samples)^a,b^; not specified (2 samples)^b^ *Ethnicity: *74% Caucasian, 22% African American^a^; not specified (4 samples)^b^ *SES/Class:* not specified (5 samples)^a,b^	*Ages: *8–17 years^a,b^ *Sample sizes:* 93–301^a,b^ *Gender:* balanced^b^; 56% male^a^; 52% female, 39% male, 8% unknown^b^ *Ethnicity:* 97% Caucasian^a^; not specified (2 samples)^b^ *SES/Class*: not specified (3 samples)^a,b^	
**PSC**—developed in 1986 (Jellinek et al., 1986)			
*23 studies (15 by developers)* a = Boothroyd and Armstrong (2010); b = Gardner et al. (2007); c = Jacobson et al. (2019); **d = Jellinek et al. (**1986**) (2 samples);** **e = Jellinek et al. (**1988**);** **f = Jellinek et al. (**1995**);** g = Jutte et al. (2003); **h = Kamin et al. (**2015**);** i = Kostanecka et al. (2008); j = Leiner et al. (2007); **k = Murphy and Jellinek (**1988**);** **l = Murphy et al. (**1989**);** **m = Murphy et al. (**1992**);** **n = Murphy et al. (**1996**);** **o = Murphy et al. (**2012**);** **p = Murphy et al. (**2016**);** **q = Navon et al. (**2001**);** **r = Pagano et al. (**1996**);** **s = Pagano et al. (**2000**);** t = Parker et al. (2019); **u = Sheldrick et al. (**2012**);** **v = Sheldrick et al. (**2013**);** w = Simonian and Tarnowski (2001)	*Ages: *under 18 years *Sample sizes:* 31–1593^b,d,h,o,^ *Gender: *balanced^b,h^; 77% male^d^; 56% male^o^; *Ethnicity: *over 90% White^b,d^; not specified^h,o^ *SES/Class:* 54% low SES^D^; 14–20% Medicaid recipients^h,o^; not specified^b,o^ *Compared reliability for children with and without physical and/or mental health disability:*^a^ Gender: 55% male; Ethnicity: 38% White, 37% Black/African American, 24% Other (mainly Hispanic); Class: all Medicaid recipients	*Ages: *0–18 years *Sample sizes:* *pediatric primary care*: 72–80,680; *other*: 117–6492 Recruited from pediatric primary care waiting rooms or well-child visits: ^d–g,i–k,m,n,p–r,u–w^ *Gender:* balanced^d–g,i,j,k,n,p,r,v,w^; 58% female^m^; 57% male^u^; not specified^q^ *Ethnicity:* 99% White^d^; 23–29% Black, Hispanic or mixed^e,k^; 74% White, 17% African American^u^; 62% White, 24% African American, 10% Asian^v^; 47% White, 53% Other^f^; 88% African American^i^; 98% African American or mixed^m^; 54% Caucasian, 46% African American^w^; 95–100% Mexican–American/Hispanic^g,j,n,r^; not specified^p^ or ethnically diverse^q^ *SES/Class:* all inner city and 45% Medicaid recipients^m^; over 70% middle/upper-middle class^e,f,k^; primarily low-income or Medicaid recipients^g,i,j,n,r^; all SES levels^d,u–w^; not specified^p,q^ Recruited from schools ^l,s^ Balanced gender^l,s^; 7% Black or Hispanic^l^; 77% African American^s^; middle to upper-middle class^l^; eligible for free breakfast/lunch^s^ Foster youth:^c,t^ balanced gender^c^, 55–60% female^t^; 49–55% White, 16–19% Hispanic, 13–16% multiracial^c,t^; no SES information^c,t^ Translation: English and Spanish^q^; 85–100% used Spanish version^g,j,n,r^	*Notes on individual studies* Studies E and K used the same data set and are counted as a single sample Study F aggregated data from 5 other studies, three of which^e,d,m^ are discussed in this review Studies U and V discuss measure development; only the validation (“replication” samples are included here)
**SDQ**—developed in 1997 (Goodman, 1997)			
*12 studies (1 by developers)* **a = Bourdon et al. (**2005**);** b = Deutz et al. (2018); c = Dickey and Blumberg (2004); d = Downs et al. (2012); e = He et al. (2013); f = Hill and Hughes (2007); g = Jee et al. (2011); h = Kovacs and Sharp (2014); i = Mason et al. (2012); j = Owens et al. (2016); k = Sheldrick et al. (2015) (2 samples)*; l = Yu et al. (2016)	*Ages:* 12–17 years^h^ (or age not specified^i^) *Sample sizes:* 65–159^h,i^ *Gender:* balanced^h,i^ *Ethnicity:* 93.2% Caucasian^h^; 47% White, 27% Black or African American, 15% two or more races^i^ *SES/Class*: 28% at/below poverty level^i^; not specified^h^ *Other demographics*: psychiatric inpatients^h^; youth in residential treatment (group home)^i^	*Ages: *4–18 years^a–c,e,g,j,k,l^ *Samples sizes:* 50–9878^a,b,c,e,g,j,k,l^ *Gender: *balanced^a,b,e,l^; 60% male^g^; not specified (3 samples)^c,k^ *Ethnicity: *95% White^j^; 65.5% non-Hispanic White^e^; 64% African American, 18% White^g^; 37% Hispanic, 34% White, 22% African American^b^; Chinese/Korean immigrant parents^l^; not specified (2 samples)^k^ but Black or African American and Hispanic households were oversampled^a,c^ *SES/Class:* 33.2% low-SES^e^; 63.5% economically disadvantaged^b^; not specified (6 samples)^a,c,g,k,l^ but 59% of mothers and 35% of fathers had at least some college education^j^ *Translation: *77.5% Chinese, 41.6% Korean, 10.9% English^l^ *Other demographics*: a, c, e and k used data taken from national household surveys**; 82% of parents were college educated^l^; foster youth, 42% had been with current foster parent for over 6 months^g^	*Children identified as “at risk” based on SES*^*d*^* or low score on literacy test*^*f*^*:* *Ages: *3–7 years^d,f^ *Sample sizes:* 298–784^d,f^ *Gender: *balanced^d,f^; 44% female^d^ *Ethnicity:* 65% Latino/a, 34% Euro-American^d^; 37% Hispanic, 36% Caucasian, 22% African American^f^ *SES/Class: *low^d^; 62% economically disadvantaged *Translation: *43% Spanish^d^; 24% bilingual or limited English proficiency, received both Spanish and English versions^f^
**TOP**—developed in 2005 (Kraus et al., 2005)			
*1 study (by developers)* Baxter et al. (2016)	*None*	Ages: 3–18 years; Sample size: 203; Equal genders. Ethnicity and class not specified	
**Y-OQ**—developed in 2001 (Burlingame et al., 2001)			
*3 studies (all by developers)* a = Burlingame et al. (2001) (6 samples)***; b = Dunn et al. (2005) (2 samples); c = Ridge et al. (2009)	*Ages:* 4–17 years^a,b^ *Sample sizes:* 61–9563^a,b^ *Gender: *balanced^b^; 58–62% male (4 samples)^a^ Ethnicity and class not specified (5 samples)^a,b^	*Ages*: 4–18 years^a−c^ *Sample sizes:* 81–1104^a−c^ *Gender:* balanced (4 samples)^a,b^; 62% female^c^ *Ethnicity: *82–92% Caucasian^a−c^; not specified (2 samples)^a^ *Class/SES:* primarily middle class^a,b^; not specified (3 samples)^a,c^	

This table reports the racial and ethnic categories used by the original study authors. “Hispanic” and “mixed race” were generally treated as non-overlapping categories (e.g. a Hispanic child would not be counted as White). FPL = Federal poverty level for that year according to the U.S. Census

* Sheldrick et al. (2015) tested ASEBA and SDQ with samples drawn from other studies, however they did not provide any information about gender, ethnicity or SES

** The 2001 National Health Interview Survey (Bourdon et al., 2005; Dickey & Blumberg, 2004) and the adolescent supplement of the National Comorbidity Survey (He et al., 2013; Sheldrick et al., 2015)

*** Burlingame et al. (2001) conducted three comparison studies using different combinations of seven separate school, community, and clinical samples

### Pediatric Symptom Checklist—PSC (n = 23 Articles; 23 Study Samples)

#### Sample Diversity

Studies included diversity across ethnicity, culture (including immigrant children), class and gender. One third (7 studies) were predominately (over 60%) White, while half were either mixed (5) or predominately African American (3) or Latino (4). Four studies did not list ethnicity. Two thirds of the studies recruited low-income or Medicaid-receiving participants (9) or used a mixed-class sample (6). Three were mostly middle class, and four did not provide information. Two studies focused on foster youth and five focused on Spanish-dominant parents. Three quarters of the community samples (14 of 18) were recruited via pediatric primary care.

Results suggest using a lower clinical cutoff for disadvantaged families (Simonian & Tarnowski, 2001) and children of Latino immigrants (Jutte et al., 2003). Murphy et al. (1996) found that Mexican immigrant parents scored their children slightly higher when answering the PSC orally than when filling out the written form, suggesting that they are more likely to describe problems verbally. Pagano et al. (1996) and Jutte et al. (2003) also validated PSC for Spanish speaking parents.

Validity results for low-income and minority children were also mixed, as discussed below. Gender results were mixed: Leiner et al. (2007) found no significant gender differences for Mexican families while Boothroyd and Armstrong (2010) found small to moderate gender effect in a mixed-ethnicity sample.

For children in foster care, PSC showed slightly lower test–retest reliability (Jacobson et al., 2019), moderate convergent validity (Parker et al., 2019), and mixed results for discriminant validity (Jacobson et al., 2019; Parker et al., 2019).

Validity for low-income and minority children is mixed: Earlier studies supported the validity of PSC with African American and low-income children (Murphy et al., 1992) and showed comparable validity and reliability compared to middle class children (Jellinek et al., 1986; Murphy & Jellinek, 1988). However, Kostanecka et al. (2008) found PSC-17’s externalizing and attention subscales to have low discriminant validity with their low-income, predominately African American sample.

#### Psychometric Properties

PSC showed high inter-rater reliability between parent and student (Murphy et al., 1989), significant correlation with parents’ reports of functioning problems (Pagano et al., 1996, 2000), moderate correlation with pediatricians’ (Jellinek et al., 1986, 1988), teachers’ (Pagano et al., 2000) and school counselors’ reports (Murphy & Jellinek, 1988), and moderate to high agreement with other standardized measures including CBCL (Jellinek et al., 1986; Leiner et al., 2007), CGAS (Jellinek et al., 1988; Murphy et al., 1992), SCARED and CDI (Gardner et al., 2007; Parker et al., 2019) and the Diagnostic Interview for Children and Adolescents Parent Report (DICA-P) (Jellinek et al., 1988).

PSC showed high specificity compared to pediatrician ratings: Jellinek et al., (1988, 1995) found that children who had experienced high stress might meet clinical criteria on PSC even when rated as functional by pediatricians. However, they also found an overall trend of pediatricians under-detecting problems when compared to child psychologists, particularly for low-income families (Jellinek et al., 1995).

Construct and discriminant validity were high (Jacobson et al., 2019) as were reliability over time and as an outcome measure (Boothroyd & Armstrong, 2010; Murphy & Jellinek, 1988; Murphy et al., 1992, 2012; Navon et al., 2001). Test–retest reliability was high for PSC-35 (Jellinek et al., 1986; Navon et al., 2001) and PSC-17 (Murphy et al., 2016) as well as the preschool PSC-18 (Sheldrick et al., 2012), and moderate for the 0–18 month Baby PSC (Sheldrick et al., 2013).

### Achenbach System of Empirically Based Assessment— ASEBA (n = 23 Articles; 25 Samples)

#### Sample Diversity

Studies included diversity across class and gender although half of the samples (12 out of 25) had larger proportions of boys. Like PSC, one third were predominately White (9 samples, 8 studies), but African American families (3 studies) and Spanish speaking Puerto Rican families (2 studies, 3 samples) were represented, as well as six studies (5 samples) using ethnically diverse samples. Four studies (5 samples) did not provide ethnicity.

Half the studies (13 studies, 14 samples) included class information. Of these, the community samples were either middle class (3 from 2 studies) or mixed (4 from 5 studies), while the samples of children receiving mental health care were predominately lower income (5 of 7). ASEBA was also tested with families with special needs adopted children (Tharinger et al., 1986) and military families (Jensen et al., 1993, 1996). Two studies validated CBCL for Spanish speaking Puerto Rican parents: Rubio Stipec et al. (1990), who used their own translation, and Bird et al. (1987a).

Konold et al. (2004) found no effect of child/parent gender, but others found that the Attention Problems subscale correlated with internalizing conditions for girls but externalizing ones for boys (Song et al., 1994), and that ASEBA had trouble distinguishing between girls' anxiety versus depression (Ebesutani et al., 2010). An early version of CBCL showed poor factor model fit for African American families (Jastrowski Mano et al., 2009) with a mismatch between parent-reported problems and CBCL’s list of problem items (Lambert et al., 2002). These issues are not mentioned in later studies and may have been fixed.

#### Psychometric Properties

Inter-rater reliability is high (Reed & Edelbrock, 1983) including between parent and clinician (Dutra et al., 2004), parent and teacher (Konold et al., 2004), parent and child (Ebesutani et al., 2011), and mother and father (Ebesutani et al., 2010; Konold et al., 2004). ASEBA shows strong correlation with relevant variables such as hospitalizations, suicide attempts, personality pathology, and family history (Dutra et al., 2004), as well as academic achievement measures (Hogez & McKay, 1986), and clinical diagnoses and history of mental health service use (Jensen et al., 1996; Rubio-Stipec et al., 1990; Tharinger et al., 1986). Jensen et al. (1996) found that while CBCL was less accurate than a diagnostic interview (DISC), it was “reasonably comparable.” There is also strong correlation with other standardized measures including BASC (Jastrowski Mano et al., 2009) and the DSM III (Tharinger et al., 1986) among others (Jastrowski Mano et al., 2009; Lambert et al., 2002; Nakamura et al., 2009; Salcedo et al., 2018; Tharinger et al., 1986). Although Reed and Edelbrock (1983) found strong correlations between TRF scores and referrals for problem behavior, Nelson et al. (2002) found low correlations with school disciplinary referrals for behavioral problems.

Discriminant validity is supported, although CBCL has higher specificity (true negatives) than sensitivity (true positives) (Rishel et al., 2005). Some early studies found correlations between subscales but they were still distinguishable (Dedrick et al., 1997; Nakamura et al., 2009), and recent research shows subscales are good to fair at distinguishing between the condition targeted by the subscale and other conditions (Ebesutani et al., 2010) although poor at more detailed distinctions such as type of anxiety disorder (Knepley et al., 2019), especially for internalizing conditions (Jensen et al., 1993).

### Strengths and Difficulties Questionnaire—SDQ (n = 12 Articles; 13 Samples)

#### Sample Diversity

Gender balance was even across most samples. Three samples were primarily White, three were ethnically mixed, and three focused on specific ethnic groups: African Americans (Jee et al., 2011), Chinese and Korean immigrants (Yu et al., 2016), and Latinos (Downs et al., 2012). The other four samples (3 studies) did not include information on race or ethnicity. Half the studies did not mention class/income (6 studies, 7 samples), the others were either mixed or predominately low-income families. One study focused on foster youth (Jee et al., 2011).

Two studies examined Spanish dominant families: Downs et al. (2012) compared English and Spanish-speaking preschoolers, while a quarter of Hill and Hughes’ (2007) parent study sample were bilingual or limited English proficiency.^6^ Only two studies recruited children receiving mental health treatment: psychiatric inpatients (Kovacs & Sharp, 2014) and youth in residential care (Mason et al., 2012). The rest used community samples, although two focused on children who were flagged as “at risk” based on SES (Downs et al., 2012) or low literacy (Hill & Hughes, 2007).

Care should be taken to use the culturally-normed cutoff scores available on the developer’s webpage, particularly with immigrant families (Dickey & Blumberg, 2004; Downs et al., 2012) as three studies found cultural differences in the parent-rated Conduct Problems and Peer Problems subscales. In a cross-national study, American parents had several items correlate more strongly with the Hyperactivity and Emotional Problems subscales compared to British parents, suggesting cultural differences in interpretations of child behavior (Dickey & Blumberg, 2004) or measure responses. Yu et al. (2016) found low reliability for Chinese and Korean immigrant parents, and low convergent and discriminant validity on the Hyperactive/Inattentive scale. Finally, Downs et al. (2012) found the Emotional Problems subscale to be suitable for Spanish-speaking U.S. preschool boys, but inadequate for girls.

For foster youth, sensitivity was high compared to CHIPS (93%) when youth and foster parent reports were combined, but lower for each one alone (54% for youth, 71% for foster parents) (Jee et al., 2011).

#### Psychometric Properties

Internal consistency was high for Total Score and moderate to high for most subscales except for Peers and Conduct and the already mentioned cultural issues (Bourdon et al., 2005; Downs et al., 2012; Yu et al., 2016). SDQ showed moderate to high correlation with CBCL (Kovacs & Sharp, 2014) over time (Mason et al., 2012) and strong correlation with reports of service use (Bourdon et al., 2005) and other variables known to be predictive of mental health problems, e.g. low SES (Bourdon et al., 2005).

Good construct and longitudinal validity (Deutz et al., 2018). Good convergent validity (He et al., 2013; Hill & Hughes, 2007) apart from the cultural issues noted. A longitudinal cohort study of first-graders showed poor discriminant validity (Hill & Hughes, 2007). Test–retest reliability is strong (Downs et al., 2012).

### Child and Adolescent Functional Assessment Scale—CAFAS (n = 4 Articles; 3 Samples)

#### Sample Diversity

Murphy et al. (1999) recruited a predominately Hispanic community sample of English and Spanish speaking low-income pre-school children enrolled in a Head Start program.^7^ Hodges and Wong (1996) recruited predominately White children of army personnel who had been referred for mental health services; they also compared ratings for fictional written patient vignettes. Bates et al. (2006) collected clinician and student ratings of individual questions items.Francis et al. (2012) compared CAFAS and GAF (the adult version of CGAS) for a multi-ethnic sample of adolescents referred for mental health evaluation, but did not present any conclusions regarding the accuracy of either measure.

#### Psychometric Properties

Inter-rater reliability was moderate to high for written vignettes for the school-age CAFAS (Hodges & Wong, 1996) and for parental reports for the preschool PECFAS (Murphy et al., 1999). There is strong correlation with reported problematic behaviors, poor academic performance, and teacher rating of psychosocial problems (Hodges & Wong, 1996; Murphy et al., 1999). The only construct validity tests were based on graduate student ratings of individual items (Bates et al., 2006). In a comparison with CAFAS, GAF identified roughly equal proportions of functional impairment for youth with externalizing versus internalizing diagnoses, while CAFAS identified twice as many externalizing cases compared to internalizing ones (Francis et al., 2012).

### Children’s Global Assessment Scale—CGAS (n = 4 Articles; 5 Samples)

#### Sample Diversity

One sample was all male, two were at least two thirds male, and two were balanced gender. Two studies (3 samples) included information on ethnicity: Francis et al. (2012) used a multi-ethnic sample to compare the older GAF version with CAFAS (see above) and Bird et al. (1987b) recruited clinic-referred and community samples of Spanish speaking Puerto Rican children. Two studies used predominately working-class samples, the others did not list such information. Green et al. (1994) extracted ratings from clinical records of psychiatric inpatients, they found different patterns from other studies (see below).

#### Psychometric Properties

CGAS showed consistently high inter-rater reliability (Bird et al., 1987b; Shaffer et al., 1983) even among raters with different types of experience, such as psychiatrists versus nurses (Green et al., 1994). It also showed high discriminant validity between clinic and community populations (Bird et al., 1987b), and between inpatients and outpatients (Shaffer et al., 1983). While Bird et al. (1987b) found high concurrent validity with CBCL, Green et al. (1994) found no significant correlation between CGAS rating and symptomatology measures (the CBCL Behavior Problems subscale) but did find strong correlations with measures of functioning (e.g. the CBCL Activities and School subscales, WISC-R IQ scale, and measures of social relatedness), and these were stronger for higher functioning children. Green and colleagues explain the discrepancy in results by arguing that their study used clinical records, in contrast to more controlled studies (such as Bird’s), which either used homogenous structured data (e.g. written vignettes or videos) or tested with raters who were not involved in the child’s treatment. In both cases, study raters would be focusing on different features than would clinicians and staff in normal practice. Additionally, they add, CBCL is normed on non-clinical samples which, by definition, would have a broader range of scores than the psychiatric inpatient samples that Green’s group looked at. Francis et al. (2012) also found discrepancies between GAF and CAFAS (discussed above) but did not investigate the cause.

### Child and Adolescent Needs and Strengths—CANS (n = 3 Articles)

#### Sample Diversity

The short form was tested with predominately Hispanic children; the other two studies were primarily (67–78%) White. Participants in one study were Medicaid recipients (Anderson et al., 2003), income status for the others was unknown. All studies focused on children receiving mental health care; there were no community comparison samples. Worth noting, Alamdari and Kelber used Y-OQ to test concurrent validity; as we will discuss below, Y-OQ has not been adequately tested with non-White children.

#### Psychometric Properties

According to Rosanbalm et al. (2016), CANS was not designed using a psychometric approach and was originally intended to be used only for individuals, not aggregated.

Three different variations of CANS were tested: a mental health scale (Anderson et al., 2003), a short form (Alamdari & Kelber, 2016), and a trauma screener (Kisiel et al., 2018). Anderson et al. (2003) found high interrater reliability between caseworkers and researchers on the mental health scale but did not address validity. The short form showed good concurrent validity with Y-OQ’s somatic and behavior dysfunction subscales (Alamdari & Kelber, 2016), while the trauma screener showed good convergent and discriminant validity when compared to TSCC-A and CBCL (Kisiel et al., 2018).

### Youth Outcome Questionnaire—Y-OQ (n = 3 Articles; 10 Samples)

#### Sample Diversity

There was little information on sample characteristics: three of the five community samples were 80% White, two were also predominately middle class. There was no information on ethnicity or class for the others. One community sample was over two thirds female, and four clinic samples from the same study were predominately male; the rest were all equally balanced.

#### Psychometric Properties

Y-OQ showed high internal consistency across community and clinical samples for Total Score, and moderate to high for subscale scores, as well as moderate to high correlation with CBCL, the Connors Parent Rating Scale (Burlingame et al., 2001), and YSR (Ridge et al., 2009). Burlingame et al. (2001) tested discriminant validity via a sensitivity analysis using a combined sample recruited from clinics, schools, and the general community. Their results suggest Y-OQ can differentiate non-clinical, outpatient, and inpatient children. Test–retest reliability was high when tested on non-clinical samples in all three studies.

### Ohio Youth Problem, Functioning and Satisfaction Scales—Ohio (n = 2 Articles; 8 Samples)

#### Sample Diversity

Both studies provided sparse information: Dowell and Ogles (2008) (2 samples) recruited predominately Caucasian families, while Ogles et al. (2001) (6 samples) provided no information on race or ethnicity. There was no information on SES or class. Three samples were predominately male, three were balanced, two did not include gender information.

#### Psychometric Properties

Internal consistency and test–retest reliability were high (Dowell & Ogles, 2008; Ogles et al., 2001), with moderate to high correlations with CBCL (parent), Vanderbilt Functioning Index (parent and caseworker), YSR (youth), and with CAFAS, CGAS and the Progress Evaluation Scales (all caseworker reports) (Ogles et al., 2001). CAFAS and CGAS both have fairly little testing with non-White children (CAFAS with Latino preschoolers and CGAS only with Puerto Ricans); both are discussed elsewhere in this paper. Moderate correlation with the BASC was found for a community sample, but no statistically significant correlation for the corresponding service client sample (Dowell & Ogles, 2008).

### Treatment Outcome Package—TOP (n = 1 Article; 1 Sample)

#### Sample Diversity

Community participants were given anonymous packets to fill out and return by mail, no details about ethnicity or class were collected. There were no studies testing TOP with children receiving mental health care.

#### Psychometric Properties

Moderate to high correlations between TOP’s subscales and equivalent CBCL and SDQ subscales; no information on reliability.

### Clinical Global Impressions Scale—CGI (n = 0 Articles)

There were no studies testing CGI on U.S. child populations, even though it is in use as a child mental health measure—it appeared in 8 studies in our exploratory scan—and has been used as a benchmark to test other measures developed for children (e.g. the Obsessive–Compulsive Inventory—Child Version, see McGuire et al., 2019). This gap in testing may be partly because CGI was originally developed for schizophrenia (Guy, 1976) and only later adopted as a general measure of functioning. Regardless of the reason, we mention it in this review because its reliability and validity need to be examined with child populations if it is to be used as a general mental health measure.

## Aggregated Results Across Measures

### Use of Measures in Research and Clinical Practice

#### Extracting Data from Clinical Records to Monitor Quality of Care

In the exploratory literature scan, at least 12 of 57 studies pulled data from existing clinical records. While these records confirm feasibility of the measures’ use in usual care practice for community-based outpatient settings, they also reveal that irregular follow-up intervals are common, for example administering only at intake and discharge. This can complicate comparing patients or aggregating results, and can also make it difficult to analyze effectiveness of care for children who have dropped out mid-treatment. Archival records therefore may not be sufficient for state or county level quality monitoring.

#### Measures that were Used as Benchmarks for Psychometrics Testing

Across the 73 psychometrics articles, five of the candidate measures (ASEBA, CGAS, CAFAS, SDQ and Y-OQ) were used as benchmarks in psychometrics tests of other measures, another indicator of their popularity. The ASEBA package appeared in 15 articles and was used as a benchmark for all the other measures except CANS. CGAS appeared in 8 articles to test CBCL, Ohio, and PSC. The other three each appeared once: CAFAS for Ohio, SDQ for TOP, and Y-OQ for CANS.^8^ All except Y-OQ have some testing with minority (African American or Latino) families, as well as with low income or working-class families.

### Breadth and Limitations of Psychometric Testing by Sociodemographic Characteristics

Examining the entire corpus of psychometric studies reveals systematic gaps in the populations with whom the measures are being tested, namely: a tendency toward male-dominated samples in clinic populations; lack of representation of Asian American, Pacific Islander, and Native American children; and lack of examination of differences across social class or SES. For information on the status of each measure, see Table 4.

**Table 4:** Summary of sample diversity by measure

	Tested across genders?		Tested across race & ethnicity?		Tested across class or SES?		Tested with non-English speaking families?	
	System ID	Community	System ID	Community	System ID	Community	System ID	Community
ASEBA	✓	✓	✓✓ (A) B L/PR M W	✓✓ B L/PR W	✓	✓	Spanish	Spanish
CAFAS/ PECFAS	Samples were over 60% male		✓ (A) (B) M W	✓ L (W)	✓	Mostly working class	No	Spanish
CANS	✓	-	✓ (B) L W	-	Medicaid eligible or not specified	-	No	-
CGAS	✓	✓	✓ (A) L/PR M (W)	✓ L/PR	Low income or not specified	Not specified	Spanish	Spanish
Ohio	✓	✓	(B) W or not specified	W or not specified	Not specified	Not specified	No	No
PSC	✓	✓	✓✓ B L W	✓✓ B L (M) W	✓	✓	No	Spanish
SDQ	✓	✓	✓ B W	✓✓ A B L W	✓	✓	Spanish	Chinese, Korean
TOP	-	✓	-	Not specified	-	Not specified	-	No
Y-OQ	✓	✓	Not specified	W or not specified	Not specified	Mostly middle class or not specified	No	No

Check mark indicates diversity across multiple published studies. Dash indicates no published studies for this population. For race and ethnicity, one check-mark indicates any psychometrics tests on racially diverse or predominately non-White samples and two check-marks indicates two or more non-White groups (not counting mixed-race) were represented

*A* = Asian or Asian American; *B* = Black or African American; *L* = Latino or Hispanic; *L/PR* = Puerto Rican Latino or Hispanic; *M* = mixed-race; *W* = White or Caucasian. A letter in parenthesis means the population was small but formed at least 15% of a study sample. American Indians, Alaska Natives, and Pacific Islanders were not represented

#### Testing by Gender

Almost all study samples^9^ (83 of 89; 93.3%) included participant gender. Of these, over half (48; 57.8%) had a roughly even gender balance,^10^ while over a third (31; 37.3%) were all or mostly male, including five all-male samples. In contrast, only four samples (4.8%) were mostly female. Two thirds (32 of 52; 61.5%) of the community (i.e. “control”) samples were gender balanced, however when examining samples recruited from clinics or other contexts where children had been referred for emotional or behavioral problems, we found that over half of them (20 of 37; 54%) were male-dominant.

#### Testing by Race and Ethnicity

While only two thirds of samples tracked participant race or ethnicity (61 of 89; 68.5%), most published studies (58 of 72;^11^ 80%) provided information for at least one sample. Of the 61 samples for which information was provided, nearly half (27; 44.3%) were predominately White, a third (19; 31.5%) were predominately non-White and a quarter (15; 24.5%) were mixed.

The most frequently listed non-White categories were African American or Black, Hispanic or Latino (sometimes as separate classifications), and mixed-race. In contrast, Asian Americans, Pacific Islanders, and Native Americans were not even mentioned in many of the studies. Only three studies had samples with more than 10% of A/PI-heritage participants: Francis et al. (2012) (17%); Nakamura et al. (2009) (14%); and Yu et al. (2016) (who focused exclusively on Chinese and Korean immigrant parents).

#### Testing by Class and Socioeconomic Status

Half of the study samples (45 of 89), and nearly half of the published studies (31 of 72, 43%), had no information on participants’ socioeconomic status, class, or household income. Of the 44 samples that did, almost half focused on working class or low-income demographics (21, 47.7%; or 23.6% of total corpus) while over a quarter used mixed-class samples (12, 27.3%; or 13.4% of total corpus). This combination of facts raises some concerns of possible selection or sampling bias, i.e. that the researchers who include information on social class tend to be those who are intentionally recruiting low-income participants or creating mixed class samples. Studies that do not report such information may be more class-homogeneous or less representative, particularly in older studies as health research has suffered from a lack of data collection on economic status or social class (see e.g. Krieger & Fee, 1994). Of course, it is impossible to say for certain since we only have access to what is in the published articles, but caution should be taken when generalizing from these studies.

#### Testing by Linguistic and Cultural Diversity

The majority (83%) of the 72 studies used the original English version of the measure. Eleven studies (for 5 measures) included Spanish-speaking families, and one study focused on Chinese and Korean-speaking immigrant parents. Table 4 presents the breakdown by measure, and the individual studies are cited in Table 3. For this review, we did not examine studies that compared how well a translation matched the original English-language measure because these would not provide information about the accuracy and quality of the measure itself. However, we encourage clinicians working with limited English proficiency families to seek out such studies (e.g. Stolk et al.’s, 2017 literature review on translations of SDQ into languages spoken by refugee families).

#### Other Participant Characteristics

Most of the measures were tested on both community samples and samples of children diagnosed with, or receiving care for, a mental or behavioral health condition. The exceptions were CANS, which was only tested on mental health care clients, TOP, which was only tested on an anonymous community sample, and CGI, which, as mentioned, was not tested on children at all. Some studies examined more specific populations such as special needs adopted children (Tyson et al., 2011) or children of military personnel (Hodges & Wong, 1996), details on these can be found in Table 3.

## Discussion

This discussion will address two issues that emerged in our study: the specific absence of Native American and Asian American families in psychometrics testing, and the more general lack of diversity testing for several measures in popular use. We will examine how cultural and linguistic differences (including those emerging from racial or ethnic, class, or geographic differences) can lead to errors on standardized measures, discuss the pros and cons of possible solutions (including norming of cutoff scores), and provide two examples of measures that were intentionally designed for Native American youth.

### Degree of Testing of Psychometric Properties Across Diverse Populations

As discussed above, published evidence supporting reliability and validity were found for all measures except CGI, however the number of studies and the diversity of participants varied widely.

The number of publications for each measure varied widely, however this number does not indicate a measure’s quality or the thoroughness of the testing. Once the initial validity and reliability tests have been published, there is little incentive to publish replicated results unless a significantly different new version is released or unless the author can provide something new that was not in the original publication, such as applying it in a different setting or with a different population.^12^ While the two measures with the most publications (ASEBA and PSC, with over 20 studies each) did have testing across a more diverse population, overall breadth of testing is more important than number of articles, and here many of the measures fell short.

Three measures (PSC, SDQ and ASEBA) were tested across diverse genders, across SES or class, and with both Latino and African American as well as White children, however none were adequately tested with Native American or US-born Asian American children. Of these three measures, only ASEBA and PSC had breadth of testing across both community populations and diagnosed (or in-care) children.

Table 4 presents a comparison of gaps in testing for each measure. The popularity of CANS in particular (used in over half of California counties, see Pourat et al., 2016a), suggest that in some cases measures might be used in clinical practice without knowing whether they are suitability to the client population.

### Underrepresented Populations: Asian Americans, Native Americans, and Immigrants

Information on Asian Americans, Pacific Islanders, and Native Americans was heavily lacking for all measures. Only three studies had over 15% Asian-identifying participants; two did not provide any data about ethnic differences, and the third (Yu et al., 2016) focused on Chinese and Korean immigrants, not US-born Asian Americans. Native Americans were not even examined.

These absences are problematic as both populations are highly vulnerable. American Indian/Alaska Native adolescents have the highest rates of depression of all ethnic groups (American Psychiatric Association, 2017). Both Native American and Asian American adolescents have rates of suicide ideation and suicide attempts that exceed those of White adolescents (US Dept. of Health & Human Services: Office of Minority Health, 2018a, b). There is also evidence of differences in symptom reports for parents of Asian and Pacific Islander ethnicity compared to non-minority parents (Okamura et al., 2016) even when child self-reports are similar, indicating a need for measurement tools tailored to these groups.

Immigrant families were also under-represented. There were a few studies involving Mexican immigrants or parents who spoke Spanish, but none with immigrants from other language groups. Currently one out of ten U.S. inhabitants (12.9%) and nearly one third of California inhabitants (27.2%) are foreign born, and that number is predicted to rise in the future (Trevelyan et al., 2016; U.S. Census Bureau, 2016), therefore this population needs further attention.

### Lack of Evidence Does Not Equal Low Quality

A brief caveat: the lack of robust testing does not indicate a measure’s lack of suitability for diverse client populations, simply that further testing is required. We encourage interested researchers to fill in the gaps discussed here, and we encourage developers to broadly pilot their measures across gender, race and ethnicity, and social class. In the next section, we briefly highlight ways a standardized measure may fall short when administered with populations for whom it was not designed.

### How Culture, Class, or Language Impact the Accuracy of Standardized Measures

It is important for care providers to be aware of how parents’ background, upbringing, and even language,^13^ can impact their responses on standardized measures and interviews, and how unintentional misreporting or miscommunication about a child’s status can cause outsized effects on children and families. Cultural and linguistic differences are not limited to immigrant parents, we can find different cultural values and dialects across classes, racial or ethnic groups, and even geographic communities within the U.S.

Misdiagnoses (“false positives”) due to unclear reporting put financial burden and emotional distress on a family in addition to lost time (Au-Yeung et al., 2018; Baker & Bell, 1999). Failure to diagnose a problem (“false negatives”)—for example because questions were not understood or symptoms were not described in a way that the clinician recognized (Bailey et al., 2009; Shen et al., 2018)—can also lead to inappropriate treatment, or even denial of services if a standardized diagnostic tool does not accurately show a child’s level of need.

To give a severe example, measurement tools that assume Euro-American childrearing practices have led to First Nations parents being judged unfit (and children removed from their homes), because they are designed to assess a two-parent nuclear family rather than an extended family of multiple primary caregivers (Choate & McKenzie, 2015).

The importance of accurate reporting becomes heightened as primary care providers are being given increased responsibility in diagnosing, referring, and sometimes even treating mental illness issues themselves (Glazier et al., 2015), especially with low income families (Hodgkinson et al., 2017). While pediatricians are adapting to meet this need (Foy et al., 2019), they do not have the same level of training as a specialist and must rely more heavily on standardized measurement tools.

#### Specific Issues When Administering Measures

Details of racial, ethnic and cultural bias in test design have been extensively discussed elsewhere (see e.g. Reynolds et al., 2021 for a historical overview) and there is also a body of literature on how to develop and adapt measures to different patient populations.

Here we would like to briefly point out some specific misreport issues, so that providers can be aware of and take these into account when administering standardized measures:

*Parents may have different benchmarks of severity* when asked to rank a behavior as “problematic” or “burdensome.” Their ratings may be affected by cultural attitudes toward certain behavioral problems (Heiervang et al., 2008) or by the level and type of caregiving support available. In such cases, a teacher report may also be of assistance.

*Parent/youth may be unfamiliar with the questionnaire structure.* Parents and youth of different cultural backgrounds or education levels may not understand or be comfortable with certain types of standardized questions, such as rating an emotion on a numerical scale (Lee et al., 2002) or grid-formatted questions (Ware et al., 1995). Some red flags to check for are skipped answers, especially if they are all the same type of question (Ware et al., 1995), or whether a parent often marks the “neutral” midpoint (e.g. 3 on a scale of 1–5) when asked for level of agreement/disagreement or how positively/negatively they feel about something (Lee et al., 2002).

*Differences in word nuance or meaning* can occur when a translation does not convey the nuance of the original language, as well as when parent and clinician (or measure developer) speak different geographic or class dialects (Epstein and et al., 2015; Leplège et al., 1998). Similarly, the same diagnostic term may have different presentations in different cultures (Haroz et al., 2017; Jani & Deforge, 2014), and folk illness categories may not translate neatly into biomedical illness categories (Flores et al., 2002).

*The diagnostic tool may not ask about (or code for) the parent’s concerns.* Connected to the previous point, social environment and community values affect children’s symptom presentation and what behaviors parents consider problematic. For example, Lambert et al. (2002) (discussed in the ASEBA section above) listed over 20 concerns that appeared in clinical case notes with African American parents but could not be coded in CBCL’s schema. They hypothesized that many of these reflect the African American community’s higher valuing of community support, mutual respect, and education.

*Parent report may differ from child’s self-report.* ASEBA, SDQ, Ohio and Y-OQ all have a child version (typically for ages 11+) to supplement the parent report. While it is unsurprising that parent/teacher reports may differ from self-reports, the level of discrepancy can vary across ethnicity and across class (Ha et al., 1998; Okamura et al., 2016).

*Clinician comprehension and unconscious bias will affect interpretation of parent concerns.* Over half of the measures in our review^14^ were designed to be completed by a clinician or social worker rather than the parent. In such cases, the accuracy of the tool is dependent on the provider being able to understand parental concerns and translate them into the standardized categories and ratings on the form. Many of the parent-report issues we discussed above also come into play in interviews with providers: perception of behavior severity, different word nuances, even parents’ level of familiarity with clinical interviews and how to describe mental health symptoms (Probst et al., 2007).

Clinician’s own biases also affect the accuracy of the report. Even well-intentioned providers may hold false beliefs or unconscious stereotypes about racial or ethnic groups (Bailey et al., 2019; Hoffman et al., 2016). Such beliefs may, on the one hand, cause providers to rank an item as less/more severe, or a symptom as less/more abnormal, for similarly presenting patients of different races. On the other hand, unawareness of real differences in cultural, social, and religious upbringing may hinder providers’ ability to recognize key symptoms when described in nonmainstream ways. For example folk explanations involving blood temperature (Elderkin-Thompson et al., 2001) or possession by evil spirits (Malgady et al., 1987) may be perceived as irrelevant or, even worse, as symptoms of psychosis.

#### Addressing Clinician Bias and Developing Cultural Awareness

Providing culturally appropriate care goes beyond merely avoiding racist or classist stereotypes. Because biases and gaps in cultural knowledge are often unconscious, the care provider must actively work at being “anti-racist” and at educating oneself about social differences (Cénat, 2020). Here are some helpful starting points for clinicians and social workers interested in this topic: Cénat (2020) gives a timely guide on how to address the needs of Black patients within the larger social context of racial violence and police brutality. McGregor et al.’s (1998) holistic approach to Hawai’ian health assessment incorporates family, community, spirituality, and relationship to the local ecology. Thyer (2015) critiques the DSM while McQuaide (1999) discusses its pros and cons in social work. Finally, Rohe (1985) reveals the impact of urban planning: how the physical layout of a city or community can benefit or impair its residents’ mental health.

### Possible Solutions for Adjusting Measures to Different Populations—and Their Down Sides

#### Norms Cutoffs and Item Weighting

A common fix is to adjust clinical cutoffs, or even individual item weights, in accordance with the baseline of a community. For example, if cultural ideas of appropriate child behavior lead to consistently higher/lower ratings of impulsiveness or aggression, these items could be re-weighted in the final score.

However, there is strong danger in this solution. Using race- or class-based scoring sheets without understanding the underlying reasons for the reported differences runs the risk of reifying harmful stereotypes about populations (e.g. as innately more aggressive or with lower self-control) rather than understanding and addressing real social inequalities (Gasquoine, 2009).

That said, SDQ and ASEBA both have publicly available data on mean scores for community populations in various countries. ASEBA’s website uses three “norm groups,” (with some countries falling into different groups for different questionnaires e.g. CBCL versus YSR). It also has a Module with Multicultural Options (MMO) supplement that allows clinicians to compare a child’s score with the grading scales for multiple countries (e.g. looking at both the immigrant parent’s home country and the family’s country of residence). SDQ’s webpage lists means by age and gender, as well as a searchable database of published research studies.

#### Using Internationally Developed Questionnaires for Immigrant Families

Providers working with immigrant families may be asking whether they should look for measures validated in the family’s country of origin. Unfortunately, this can be equally problematic, as immigrant children are not raised in the same social environment as their cousins abroad. For example, Shen et al. (2017) developed and piloted a set of scales to measure anxiety, depression, and school problems in Chinese middle school students, but because their tool is designed for children immersed in China’s intense examination-oriented education system, it is not useable for Chinese-immigrant adolescents attending U.S. schools.

#### Creating Mental Health Measures for Minority Youth, Two Examples for Native American Youth

Finally, we offer two examples of mental health measures that were designed for, and tested with, Native American and First Nations youth. While these do not fit our original criteria (one is outside our age range, the other does not provide an overall mental health score), they may be of use as supplements to clinicians. Additionally, the details of their development may serve as models for researchers.

The *Life Trajectory Interview for Youth* was designed to “address gaps in our understanding of the links between large-scale structural conditions and social processes and individual outcomes such as mental health.” It was developed and pilot tested on a 60% Anglo/40% Cherokee sample of young adults (aged 19–24) living in western North Carolina. The measure covers four domains: life course milestones, life course barriers, social affordances, and material goods (Brown et al., 2006).

The *Cultural Connectedness Scale (CCS)* was designed for and piloted with Canadian First Nations, Métis, and Inuit youth (Snowshoe et al., 2015). Although not itself a mental health scale, its developers showed a link between cultural connectedness and mental wellness for these population (Snowshoe et al., 2017).

#### Use of Appropriate Benchmarks When Testing or Developing Measures

As noted earlier, several of the measures we reviewed (particularly CBCL) were used to test validity or correlations in psychometric tests of others measures. CBCL was the most widely used and it is a well-established and broadly tested measure. While we have discussed the importance of using diverse and representative study samples, it is also important for measure developers and researchers to beware of hidden biases or disparities within the benchmarks being used with these populations. For example, several of the studies reviewed here tested for correlations between a measure’s report of behavioral problems and poor academic performance (e.g. Hill & Hughes, 2007; Hodges & Wong, 1996) or school disciplinary referrals (e.g. Hill & Hughes, 2007; Hodges & Wong, 1996; Hogez & McKay, 1986; Murphy et al., 1999; Nelson et al., 2002; Reed & Edelbrock, 1983). Children of low SES families and minority children are at disproportionate risk of both of these, particularly African American boys (Heath, 1982; Skiba et al., 2002).

### Scope and Limitations of this Review

Because the original goal of this project was to find candidate measures suitable for use in California DHCS outpatient child mental health care, we narrowed the scope of our review in the following ways: (1) The initial five-year exploratory search focused on English-language articles and only public or community clinics; this may have under-represented some measures’ popularity internationally or in other systems of care. (2) We only examined psychometrics tests on U.S. populations and (3) excluded those focusing on treatments or conditions which would not be covered in DHCS outpatient settings (e.g. substance abuse or skills training for autism). (4) Because we were interested in the reliability and validity of the measure itself, we did not examine studies that only compared the fidelity of a translation to the English version, but we did include non-English translations that were tested against independent metrics.

As noted, sample representativeness was examined for gender, race and ethnicity, and class/SES, as these were the three variables reported in most studies. While other participant characteristics such as adoption or type of family (e.g. foster families or same-sex parents) were not systematically compared, they are listed in Table 3 and in the sections focusing on individual measures.

## Conclusion

By laying out the available and missing information about these ten clinical outcome measures, our goal has been to assist practitioners, researchers, and legislators in selecting appropriate standardized measures that have been tested and normed on samples that resemble their own client populations. In the process, we discovered that some popular measures lacked breadth of testing on diverse patient and community populations. Therefore, a second emerging goal of this paper has been to give clinicians insight into how cultural and linguistic differences (including those between racial, ethnic, and class groups) can impact measurement reports.

Testing with Asian American, Pacific Islander, and Native American families should be a high priority, as well as comparisons across classes. Further testing with immigrant families of various backgrounds is also needed. Because measures such as CANS, Y-OQ, and Ohio are already in wide clinical use, patient records may yield a good source of data, although we have discussed some of the caveats of using them.

Finally, researchers who are in the process of creating or adapting their own measures are encouraged to include these under-examined populations starting from the earliest stages of development and pilot testing.

## Supplementary Information

Below is the link to the electronic supplementary material.

Supplementary file1 (DOCX 27 kb)

## Footnotes

1 U.K. and Australian CAMHS (Child and Adolescent Mental Health Services) and CYMHS (Child and Youth Mental Health Services) programs were included as a type of community mental health care.

2 This count does not include measures of treatment alliance, level of service use, or parenting outcomes. Qualitative measures such as semi-structured interviews and goal-tracking tools are also not counted since they cannot be aggregated or compared across patients.

3 These were: HoNOSCA (Health of the Nations Outcome Scale for Children and Adolescents), which has not been not validated for U.S. children; CIS (Columbia Impairment Scale), which is only for children 9 years and older, and GAF (Global Assessment of Functioning, formerly GAS) which was dropped in favor of the child-specific C-GAS.

4 Searches used the topic search string: TS = ([full name of measure] OR [abbreviation]) AND TS = (psychom* OR reliability OR validity).

5 All but one of these studies used the parent report Child Behavior Check-List (CBCL); 4 studies combined the CBCL with one of the other reports in the Achenbach package: either the Youth Self Report or the Teacher Report Form.

6 These parents were mailed both Spanish and English versions of SDQ but the authors did not report how many parents chose the Spanish version.

7 Head Start is a program run by the U.S. Department of Health and Human Services. It funds local community programs for low income families with children under 5 years old, in order to promote school readiness, children’s development, and family wellbeing.

8 Two additional studies compared measures without using one as a benchmark for the other: (Francis et al., 2012) compared CAFAS and GAF while (Sheldrick et al., 2015) compared SDQ and ASEBA.

9 Several studies included multiple different participant samples with different demographics. See Table 3 for further details.

10 “Roughly balanced” was defined as no more than 55% of a single gender (including any missing data).

11 One study did not conduct any tests with families (Bates et al., 2006).

12 Examples of such specific scopes include: the child welfare system (Parker et al., 2019), pediatric primary care (Pagano et al., 1996), children of immigrants (Leiner et al., 2007), and adopted children with special needs (Tyson et al., 2011). Others are listed in Table 3.

13 Patients with other communicative or social stressors also face similar risks, see e.g. Au-Yeung et al. (2018) on mental illness misdiagnosis with autistic patients.

14 CAFAS, CANS, CGAS and CGI are provider-only. Ohio and Y-OQ have provider, parent, and child versions.
