# Supplementary material for: Overview of Ten Child Mental Health Clinical Outcome Measures: Testing of Psychometric Properties with Diverse Client Populations in the U.S
Source: Adm Policy Ment Health. 2021 Sep 5;49(2):197–225. doi: 10.1007/s10488-021-01157-z (PMC8850232; doi:10.1007/s10488-021-01157-z)
Supplement: Supplementary file 1 — Supplementary file1 (DOCX 27 kb) [file 10488_2021_1157_MOESM1_ESM.docx]

Supplemental Table 1. Inclusion and exclusion criteria for the exploratory literature scan to identify child mental health outcome measures used in community-based treatment settings.

|  | **Criteria** | |
| --- | --- | --- |
| **Topic** | **Inclusion** | **Exclusion** |
| **Publication Characteristics** | Published 2010-2015 (inclusive) in a peer-reviewed journal AND obtained through PubMed or Scopus AND has an English language abstract | Abstract does not list the measures used to assess outcomes AND English-language text is not accessible through the authors’ university |
|  | If full text was not available through the university (or not in English), only information from the abstract was compiled | |
| **Setting or Location** | Outpatient mental health programs  Both USA & international settings | Non-clinic interventions (e.g. school, home)  In-patient psychiatric unit care  Pediatric primary care  Non-diagnosed (community) populations |
| **Target Population** | Child is the primary recipient of mental health care  Age ranges:   - 0-18 years - Pediatric/adolescent studies that expand the age range to 21years (e.g. 13-20 year old adolescents) | Child is not the primary recipient of the intervention (e.g. parent/teacher training)  Child does not have a mental health diagnosis (e.g. quality of life outcomes for children with physical disabilities)  Study tracks adult outcomes only (e.g. of abuse as a child).  Age ranges:   - >18 years (including young adult studies) - ≥16 years if classified as adults |
| **Follow-up Time Points** | Measure was used to track change in child symptoms or functioning across 2 or more time points (regardless of time interval)^a^ | Measure was only used for screening or to determine study eligibility |
| **Type of Measure** | Measure yields one or more numerical scores OR ranks patient status on one or more standardized scales | Measure results are descriptive or otherwise cannot be compared between patients (e.g. individualized treatment plans)  Measure is custom-designed by researchers but not adequately described in the article (e.g. only described as "a survey") |
| **Types of Clinical Outcome** | Measure tracks either:   - Symptoms: mental health problems or psychiatric diagnoses that meet medical necessity criteria for outpatient Medicaid-funded mental health care OR - Functioning: indicators of functioning (socially, at home or academically); strengths and weaknesses | Measure tracks either:   - Other parts of the treatment process (e.g. therapist alliance; parent satisfaction) OR - Conditions that do not fall under the EPSDT program, including: intellectual disabilities; developmental or language delays; neuro-developmental issues; brain injury; autism; substance use/abuse (alcohol, drugs, smoking, marijuana) - Achievement of customized milestones set by clinician and patient/family |
| **Types of Effectiveness Trials** | Care model redesign  Community-based psychosocial interventions  Comparisons of two different interventions, or a novel intervention versus usual care | Clinical drug trials OR studies testing only medication efficacy  Proposed studies for which data has not yet been collected^b^ |

EPSDT=Early Periodic Screening, Diagnosis and Treatment, a Medicaid benefit for all enrollees under the age of 21 years

^a^ Tracking could consist of baseline/followup or comparing follow-ups at multiple time points (e.g. Clarke et al. 2015). Studies were included if they used two different versions due to child ageing out of the first one. ^b^ Descriptions of proposed studies were not included as they do not provide evidence that a measure can be successfully implemented with families (feasibility).

Supplemental Table 2. Inclusion and exclusion criteria for systematic literature review of psychometric properties of candidate measures.

|  | **Criteria** | |
| --- | --- | --- |
| **Topic** | **Inclusion** | **Exclusion** |
| **Publication Characteristics** | Published in peer reviewed journal AND obtained through one of the following:   - Web of Science database search - cited on vendor's webpage - recommended by DHCS Subject Matter Experts - cited in a literature review found through one of the above methods   There were no limits by publication date. | Unpublished or non-peer reviewed sources (e.g. conference paper or user manual)  Abstract does not adequately describe study results AND English-language text is not available through the authors’ university |
| **Geography** | Includes (but not limited to) U.S. populations  For multi-country studies, U.S. data were extracted when possible | Only examines non-U.S. populations |
| **Sample Characteristics** | Ages 0-21 years  General population (undiagnosed children) OR general (not diagnosis-specific) studies of children receiving mental health care | Only covers ages > 18 years  Focuses on physical health conditions (e.g. diabetes, chronic physical illness)  Exclusively examines a single mental health diagnosis (e.g. depression) |
| **Study Characteristics** | Examines reliability and/or validity using an external benchmark (another standardized measure, clinician diagnosis, teacher report of behavioral problems, etc.) | Only compares different versions of the same measure (e.g. parent versus child report; translation versus original English measure)  Only tests feasibility of administering the measure |
